# Integrating *In Silico* and *In Vitro* Approaches to Screen the Antidiabetic Properties from *Tabernaemontana divaricata* (Jasmine) Flowers

**DOI:** 10.1155/2022/4616815

**Published:** 2022-05-31

**Authors:** Saima Muzammil, Rahat Andleeb, Sumreen Hayat, Muhammad Umar Ijaz, Asma Ashraf, Nimrah Zafar, Shabana Naz, Mubashera Shaheen

**Affiliations:** ^1^Department of Microbiology, Government College University, Faisalabad, Pakistan; ^2^Department of Zoology, Government College University, Faisalabad, Pakistan; ^3^Department of Zoology, Wildlife and Fisheries, University of Agriculture, Faisalabad, Pakistan

## Abstract

The purpose of this study was to assess different *in vitro* biological activities such as phytochemical constituents, enzymatic antioxidant status, cytotoxicity through hemolytic activity, and antidiabetic potential of plant methanolic extract through glucose uptake by yeast cells. Further, using *in silico* approach by the *SwissADME* technique the drug-likeness rules for bioactive components were characterized, while potential interactions were identified via molecular docking of a ligand with target proteins by GOLD 5.3.0. The results showed that *T. divaricata* was rich in TPC and TFC, i.e., 62.32 ± 4.02 and 24.53 ± 0.61, respectively, and the cytotoxic potential was 10% towards human RBCs, while protein estimation revealed the presence of protein in the extract, which was 22.82 ± 4.6. DPPH assay in comparison with ascorbic acid and several enzymatic assays, such as CAT, SOD, and POD, showed maximum antioxidant potential, i.e.,15.9 ± 2.33%, 65.57 ± 13.4%, 3.02 ± 3.4, 15.87 ± 0.5, and 0.74 ± 0.2, respectively. Glucose uptake by yeast cells, i.e., *α*-amylase and *α*-glucosidase, showed a maximum antidiabetic potential such as 75.11 ± 1.44%, 41.81 ± 3.75%, and 35.9 ± 1.24%, respectively. Our results indicate that the methanolic extract of *T. divaricata* has antioxidant potential and inhibits *α*-amylase and *α*-glucosidase activity and possesses maximum antidiabetic potential. The results provide scientific proof that the medicinal plant being studied is a powerful source of natural antioxidant, antidiabetic, and medicinally significant substances. *In silico* study, using a molecular docking, unveiled that two compounds showed good interactions with 5kzw protein with considerable binding affinities and fulfilled docking parameters. It may conclude that *T. divaricata* is an important vegetable with a potent source of natural antioxidants and antidiabetic activity justifying its traditional use in green therapeutics.

## 1. Introduction

Diabetes is a metabolic disorder characterized by a high level of glucose in the bloodstream triggered by inadequate insulin output or insulin activity [1]. Diabetes is a dynamic chronic disease needing ongoing medical care with harm reduction approaches within glycemic control [2]. Polyuria, polydipsia, dry mouth, itchy skin, blurry vision, nausea, and exhaustion are the typical physical symptoms of DM [3]. Diabetes accounts for over 3.8 million deaths annually and is therefore the fifth leading cause of mortality [4]. This disease is becoming disastrously underdeveloped (low-income) countries [5]. Pakistan is amongst the ten countries that are expected to have high figures of people with diabetes by 2030 [6].

Diabetes increases the risk of several health problems and has a severe effect on the eye, kidney, foot, blood pressure, etc. [2]. *α*-Amylase involves the starch hydrolysis into the small scraps of sugar [7]. During diabetes, ROS are induced and cause *β*-cell glucose toxicity [8]. Cytotoxic T lymphocytes (CTLs) are one of the types of T cells, and CTLs are triggered to clear cells associated with the virus [9].

Oxidative stress is caused by an increase in reactive oxygen species, which is the primary cause of diabetes and can have serious consequences. Enzymes including superoxide dismutase, glutathione, peroxidase, and catalase are involved in enzymatic defense systems [10, 11]. The peroxidases are a family of enzymes with the ability to oxidize different substrates using H_2_O_2_ [12]. Antioxidants (e.g., superoxide dismutase (SOD), reduced glutathione (GSH), and other antioxidant enzymes) are generally providing tools to investigate the stress-related diabetes [13]. Several natural antioxidants are present in plants, and usually, these are vitamins C and E, tannins, and flavonoids. These antioxidants have the competency to sustain or uphold *β*-cell administration, and in this way, they could diminish the glucose level in the blood. It has been found that medicinal plants, e.g., *Coriandrum sativum, Fraxinus excelsior, Casearia esculenta, Caesalpinia bonducella, and Biophytum sensitivum,* are more cost-effective, have fewer side effects, and are more persuasive in curing diabetes mellitus than conventional drugs [14].

Due to the high cost of pharmaceuticals, typical and traditional plants could be used to cure different diseases as 70 to 80% of the developing world depends on them [15]. For this purpose, 800 plants could be considered for their antidiabetic potential [16]. A plant species known as *Tabernaemontana divaricata* (TD) belongs to the family *Apocynaceae*, locally acknowledged as Tagar/Chandni/Crepe Jasmine. In contrast to *Staphylococcus aureus* and *Escherichia coli, Tabernaemontana divaricata* flower extract has strong antibacterial activity [17].

In silico approaches to the drug design has the benefit of reducing the time and expense of developing new targets. *In silico* methods that can describe interacting molecules and predict three-dimensional (3D) structures have been used to solve several biological problems. In this context, this study aimed to evaluate the phyto-components, total phenolic content (TPC), total flavonoid content (TFC), and *in vitro* biological properties such as DPPH (2,2-diphenyl-1-picrylhydrazyl) free radical scavenging, enzymatic antioxidant activities, hemolysis, and antidiabetic potentials of methanolic extract of *T. divaricate* flower. Further, to develop effective inhibitors, *in silico* analysis was performed to determine the interaction of identified antidiabetic compounds with the target protein.

## 2. Material and Methods

### 2.1. Determination of Total Phenolic and Flavonoid Contents (TPC and TFC)

By following the protocol of Singleton et al. [18], the TPC of methanolic extract of *T. divaricata* was evaluated. The absorbance for the mixtures of solutions was measured at 760 nm with gallic acid as a standard, and TPC results were expressed as milligram gallic acid equivalent per gram dry weight (mg GAE/g dw) in triplicates.

The procedure of Bao et al. [19] was followed to calculate the total flavonoid content (TFC) of *T. divaricata*. Eventually, the absorbance of the mixtures was measured against a reagent blank at 510 nm and TFC was noted in triplicate samples and presented as milligram quercetin per gram dry weight (mg QE/g dw).

### 2.2. Antioxidant Activity Assays

#### 2.2.1. DPPH Free Radical Scavenging Assay

By following the protocol of Gyamfi et al. [20], the free radical scavenging capability of *T. divaricata* flower methanolic extract was evaluated against free radicals of DPPH. Ascorbic acid was used as a standard. The following formula was used to calculate the percentage inhibition of plant extract against DPPH free radicals:

(1) $$\begin{array}{c} \text{Inhibition percentage of extract}=\left[\frac{AB-AS}{AB}\right]\times 100, \end{array}$$

where AB is the absorbance of blank and AS is the absorbance of the sample.

#### 2.2.2. Enzymatic Antioxidant Activities

The method proposed by Mohebbi et al. [20] was performed to measure thecatalase (CAT) activity. In this method, 0.1 mL of *T. divaricata* extract was added in 0.05 M K_2_HPO_4_ and 1.4 mL hydrogen peroxide (H_2_O_2_), which was taken as a substrate, and catalase enzymes were added for the decomposition of H_2_O_2._ The decomposition was detected using a UV spectrophotometer (UV-1601, Shimadzu, Germany) by calculating the reduction in the absorbance for 5 min at 240 nm. The results of this activity are denoted as *μ*M of consumed H_2_O_2_/min/mg of protein. Similarly, peroxidase (POD) activity was checked in which guaiacol was used as hydrogen. The experiment was carried out by measuring the difference at 470 nm for 1 minute. The enzymatic activity was described as a unit (one activity unit defined as absorbance at 470 nm changes 0.001 per min) [21]. The rate of inhibition in the photoreduction of nitroblue tetrazolium (NBT) by the mean of superoxide dismutase (SOD) enzymes was calculated to determine SOD operation. The reaction mixture used in this activity has 50 mM sodium phosphate buffer of pH 7.6, 50 mM sodium carbonate, 0.1 mM EDTA, 50 *μ*M NBT, 12 mM·L-methionine, 10 *μ*L riboflavin, and 100 *μ*L crude extract within an exact having 3.0 mL volume. On the other hand, the control reaction was preceded in the absence of extract. The SOD behavior was measured by exposing the following reaction mixture to white light at room temperature for 15 minutes. After incubation for 15 min, the absorbance was noted at 560 nm with the help of a spectrophotometer [22].

### 2.3. Cytotoxicity through Hemolytic Activity

To estimate the hemolytic activity of plant extract against human red blood cells (RBCs), the method of Powell et al. [23] was followed. PBS was used as the negative control, while 0.1% Triton X-100 was served as a positive control. The absorbance at 576 nm was noted.

(2) $$\begin{array}{c} \%\text{Hemolysis}=\frac{\text{Absorbance of sample}-\text{Absorbance of negative control}}{\text{Absorbance of positive control}}\times 100. \end{array}$$

### 2.4. Antidiabetic Assays

#### 2.4.1. *α*-Amylase Inhibition Assay

*α*-Amylase inhibitory activity of *T. divaricata* flower extract was assessed by following the procedure of Shai et al. [24]. Various concentrations of extract were incubated in phosphate buffer of 0.1 mol/L at pH 6.8 and 2 U/mL porcine pancreatic amylase 500 *μ*L for 20 minutes at 37°C. 1% starch of 200 *μ*L was mixed in phosphate buffer of 0.1 mol/L with pH 6.8 and added to mixtures of extract. After incubation at 37°C for 1 h, 1 mL of color reagent (3,5dinitrosalicylic acid) was added to the mixtures, and finally, the absorbance was recorded at 540 nm after boiling this mixture for 10 minutes. Control was without inhibitor, and the percentage of inhibitory efficacies of the extract was measured using the following formula:

(3) $$\begin{array}{c} \alpha -\text{amylase inhibition}\left(\%\right)=\frac{\;A540_{\text{control}}-A540_{\text{sample}}}{A540_{\text{control}}}\times 100. \end{array}$$

#### 2.4.2. Intestinal *α*-Glucosidase Inhibitory Assay

The inhibitory capability of *α*-glucosidase was evaluated by following the procedure of Ademiluyi and Oboh [25] with some modifications. Different concentrations of the extract were mixed with a 100 *μ*l solution of 1.0 U/mL *α*-glucosidase and phosphate buffer whose pH was adjusted to 6.8, and the mixtures were incubated for 15 minutes at 37°C. After this, 5 mmol/L solutions of PNPG with the amount of 50 *μ*L in phosphate buffer 0.1 mol/L (pH 6.8) were poured into the solutions and incubated again for 20 minutes at 37°C. The absorbance of the control and sample was measured at 405 nm, and the percentage inhibitory activity of glucosidase was calculated using the following formula:

(4) $$\begin{array}{c} \text{Percentage inhibition}=\frac{A_{\text{control}}-A_{\text{sample}}}{A_{\text{control}}}\times 100, \end{array}$$

where *A*_control_ is the absorbance of the mixture without extract and A_sample_ is the absorbance of the mixture with the extract.

#### 2.4.3. Glucose Uptake in Yeast Cells

The protocol of Cirillo [26] was used to perform glucose uptake in yeast cells. Suspension of yeast (1%) was centrifuged for 5 min at 4200 rpm. By taking supernatant 10% *v/v* yeast cells, the suspension was prepared. 2 mg of an extract was dissolved in 1 mL of DMSO. These mixtures were then added in the different molar concentrations of 1 mL solution of glucose and at 37°C incubated for 10 min. For starting the reaction, 100 *μ*L of suspension was added to this mixture, then vortexed, and incubated again at 37°C for 60 min. The mixture was then centrifuged at 3800 rpm for 5 min, and for the estimation of glucose, the absorbance was taken at 520 nm. The percent increase in glucose uptake was calculated using the following formula:

(5) $$\begin{array}{c} \text{Increase in percentage of glucose uptake}=\frac{A_{\text{control}}-A_{\text{sample}}}{A_{\text{control}}}\times 100. \end{array}$$

The control consisted of all the reagents except plant extract, and metronidazole was used as a standard drug.

### 2.5. *In Silico* Study

#### 2.5.1. SwissADME Analysis

Fourteen important bioactive compounds in the methanolic extract of *T. divaricata* flowers were selected due to their bioactive potential towards antitumor, antibacterial, and anti-inflammatory properties and to give better pharmacological activities [27–29]. These compounds, enlisted in Table 1, are subjected to a theoretical *in silico* ADME prediction study using the Web tool, SwissADME (http://www.sib.swiss). To predict suitable properties, 2D structural models of analyzed compounds were drawn in SDF format and transferred to the simplified molecular-input line-entry system (SMILES) format. The SwissADME server tool was used to measure physicochemical properties of the compounds such as molecular weight, number of hydrogen bond acceptors, number of hydrogen bond donors, number of rotatable bonds, molar refractivity, lipophilicity (ALogP), and topological polar surface area (TPSA). The drug-likeness efficiency of the selected compounds was examined for first-round screening using Lipinski, Ghose, and Veber rules based on their physicochemical properties [30–32], and then, pharmacokinetics and medicinal properties were also examined.

Drug-likeness, bioavailability, and pharmacokinetic abilities are important parameters to predict the active potential molecules for the purpose of drug development. In the clinical trials, many potent medicines fail in the drug development phase because of their weak abilities. To ensure that the bioactive compounds of *Tabernaemontana divaricata* have drug-like characteristics, all of the compounds in this study were subjected to ADME and prediction experiments before molecular docking.

#### 2.5.2. Molecular Docking Analysis

The compounds that went through the filter were docked with the target protein, resulting in improved docking studies that predicted the potential binding of inhibitor to the protein. According to the results of SwissADME, 9 of 26 compounds were chosen to describe the major compounds contained in the extracts. The three-dimensional structures of compounds and target protein were calculated using the Protein Data Bank (PDB) database. GOLD version 5.3.0 and BIOVIA Discovery Studio were used to perform docking calculations (http://www.3dsbiovia.com/) for designing and visualization [33].

*(1) Protein Preparation for Dockings*. For the molecular docking studies of the synthesized compounds, GOLD docking software version 5.3.0 was used. Protein Data Bank was used to obtain the coordinated crystal structure of the 5kzw protein (http://www.rcsb.org/pdb/home/home.do) [34] and saved in PDB format. Essential hydrogen atoms were added with the aid of GOLD. GOLD score is a function that mimics a molecular mechanism and has been optimized for calculating ligand-binding positions [35]. Affinity (grid) maps of 10 Å and grid points with 0.75 Å spacing were generated [36].

*(2) Ligand and Energy Minimization*. For ligand's energy minimization, the ChemDraw Ultra 12.0 and Chem3D Pro were used. The ligand atoms were given the Gasteiger partial charges. Rotatable bonds were described by merging nonpolar hydrogen atoms. The ligands were obtained from PubChem (https://pubchem.ncbi.nlm.nih.gov) [37]. GOLD takes into account the degree of freedom in the binding site that corresponds to the reorientation of hydrogen bonds of donor and acceptor groups. Despite accounting for a small fraction of the total conformational space available, this degree of freedom accounts for a significant difference in binding energy values [38].

*(3) Ligand-Protein Docking*. For predicting the binding affinities of a variety of ligands, molecular docking protocols are commonly used [39]. The parameters of molecular docking software were used to conduct the experiments (https://www.ccdc.cam.ac.uk/). The Lamarckian genetic algorithm (LGA) and the Solis and Wets local search method were used to simulate docking [40]. The initial positions, orientations, and torsions of the ligand molecules were chosen at random. Each docking experiment was divided into ten separate runs, each of which was set to end after a maximum of 1.5 energy evaluations.

### 2.6. Statistical Analysis

The experimental results were performed in triplicate, and the data were expressed as mean ± S.E. One-way analysis of variance (ANOVA) was done for statistical analysis of data employing SPSS version 22.0. For statistical significance, *p* < 0.05 was considered. Using GOLD version 5.3.0 and BIOVIA Discovery studio visualizer (http://www.3dsbiovia.com), docking calculations were carried out.

## 3. Results

### 3.1. Phytochemical Assays

The estimated total phenolic content of *T. divaricata* methanolic extract ranged from 35.62 ± 5.31 to 62.32 ± 4.02. The estimated total flavonoid content of *T. divaricata* flower extract ranged from 13.95 ± 1.33 to 24.53 ± 0.61. The maximum quantity of total phenol and flavonoid was 1 mg, while the minimum was 31 *μ*g as shown in Table 2.

### 3.2. Antioxidant Activities

Extract antioxidant activity was measured in comparison with ascorbic acid. The measured antioxidant potential ranges from 1.49 ± 0.33 to 15.9 ± 2.33, while ascorbic acid was 1.15 ± 0.58 to 65.57 ± 13.4. The antioxidant potential of the extract was less than ascorbic acid at maximum concentration; however, the potential was improved at lower concentration in comparison with ascorbic acid as shown in Table 2.

#### 3.2.1. Enzymatic Antioxidant Activities

Enzymatic antioxidant activities by *T. divaricata* such as catalase (CAT), superoxide dismutase (SOD), and peroxidase (POD), as shown in Table 3, were 3.99, 1.27, and 23.80 U/mg protein, respectively. The free radical scavenging, along with an increase in physiological antioxidants (e.g., CAT, SOD, and POD), was possibly due to the presence of the phytochemicals of this plant.

### 3.3. Cytotoxicity through Hemolytic Activity

Hemolytic activity of any compound is a sign of common cytotoxicity towards normal healthy cells. In this research, the extract exhibited 10% cytotoxicity towards human erythrocytes as shown in Table 3. Such findings demonstrated that if medicinal formulations from this plant are used at low concentrations, the parameters of the RBC membrane will not change. Further research is essential on this medicinal edible plant in the context of drug development and discovery.

### 3.4. Antidiabetic Potential

#### 3.4.1. *α*-Amylase and *α*-Glucosidase Inhibitory

The estimated *α*-amylase concentration range of *T. divaricata* flower extract was 16.76 ± 3.97 to 41.81 ± 3.75. The average values of *α*-glucosidase inhibitory activity ranged from 17.88 ± 1.40 to 35.9 ± 1.24. The inhibition potential of both enzymes was maximum at 200 ug/mL and minimum at 50 ug/mL as shown in Table 4.

#### 3.4.2. Glucose Uptake by Yeast Cells

**Figure 1:**
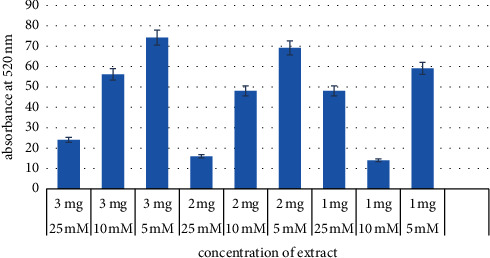
Percentage of glucose uptake by yeast cells.

The uptake of glucose was evaluated by incubation of yeast cells in different molar concentrations of glucose and *T. divaricata* extract. At 5, 10, and 25 mM glucose concentrations with 1, 2, and 3 mg/mL concentration of *T. divaricata* extract, the results showed that by rising the extract concentration the uptake of glucose percentage was increased, while it showed an inverse relation with the glucose molar concentration as described in detail in Figure 1, which shows the maximum absorbance at 5 mM of glucose and 3 mg/mL concentration of extract.

### 3.5. *In Silico* SwissADME Analysis

Due to weak drug-likeliness and pharmacokinetic characteristics, many potent drugs fail in clinical trials or later stages of drug discovery. All of the compounds in this sample were subjected to drug-likeness and ADME prediction tests before molecular docking to ensure that they had drug-like characteristics.

#### 3.5.1. Physicochemical Properties

A drug's physicochemical properties have a significant effect on its metabolic destiny in the body. The results from Table 1 showed that the molecular weight of all compounds met the criterion (which should be ≤ 500 g/mol) except D-glucopyranoside (MW 504.44 g/mol) in accordance with one of the criteria laid down in the Lipinski rule of five. Of all the studied compounds, 17 compounds had less than 10 rotatable bonds, and others had more than 10, which is not acceptable. Further, the molar refractivity of all compounds was within the acceptable range (40 and 130) except squalene, 1-heptatriacotanol, and methyl ester, and these three compounds satisfy the criteria for oral bioavailability.

TPSA is another key parameter correlated with the drug's bioavailability. High oral bioavailability for passively absorbed compounds has TPSA < 140 Å2. Table 1 reveals that all the selected compounds were found to be polar with TPSA values ranging from 0.00 Å2 to 80.48 Å2 except D-glucopyranoside, desulphosinigrin, and lactose, which had the highest TPSA (>140 Å2). High solubility can facilitate complete absorption of the administered through oral administration, while low solubility limits the drug absorption in the gastrointestinal tract [41]. Table 1 shows that all of the tested 26 compounds have good to moderate water solubility, with a log S value between −0.18 and −5.6, which may promote good oral adsorption.

#### 3.5.2. Pharmacokinetic Properties

Interestingly, except for three compounds, as shown in Table 5, all were observed with high intestinal absorption and thus could penetrate very easily through the intestinal lining and be accessible to the cell membrane. To meet their molecular target, drugs that function in the central nervous system (CNS) need to move through the blood-brain barrier (BBB). However, for drug molecules with a peripheral target, little to no BBB permeation may be needed to prevent side effects on the central nervous system [42].

The blood-brain barrier (BBB) permeation expresses the relative affinity of the drug for the blood or brain tissue. Table 5 indicates that 15 compounds are estimated to have no penetration of the blood-brain barrier, and thus, the chance of CNS side effects is expected to be absent. P glycoprotein (P-gp) plays a significant role in protecting the central nervous system from xenobiotics [43]. The predicted outcome shows that only 9 of 26 compounds are P-gp substrates and do not cause phospholipidosis. The other 17 compounds on the other hand are non-substrates of P-gp and are therefore required to induce phospholipidosis on the phenyl ring. The cytochrome P450 (CYP) superfamily is critical in drug removal through metabolic biotransformation [44]. The less skin permeant the molecule is, the lower the log Kp (in cm/s) is. It is found that log Kp measurements of all the compounds evaluated are within the limits (−8.0 to −1.0) except D-glucopyranoside, desulphosinigrin, and lactose [45].

#### 3.5.3. Lipophilicity and Drug-Likeness

The result of Table 6 showed that the log *P* values of all the compounds except benzene dicarboxylic acid, methylnonadecane, phthalic acid, 1-heptatriacotanol, and cyclopropane tetradecanoic acid. The selected compounds were found to be within the limits, i.e., between 0.7 and  + 5.0, indicating that they should have strong permeability and oral absorption. Drug-likeness qualitatively tests a molecule's likelihood of being an oral drug candidate for bioavailability [45].

#### 3.5.4. Rule of Five by Lipinski

As per Lipinski's rule of five [31], the drug is likely to be produced as a prospective oral drug if the applicant violates none or less than one of the following four conditions. The Abbot bioavailability score (BAS) is a rule-based semi-quantitative score that relies on a total charge, TPSA, and the Lipinski filter violation that distinguishes four compound groups. All of the selected molecules have a bioavailability score of 0.55, except for tetrazole, octadecadienoic acid, n-hexadecanoic acid, and lactose, indicating a chance of becoming the oral drug candidates [45].

In this investigation, 17 of 26 of the selected compounds did not pass ADME screening, leaving 9 candidate compounds for the molecular docking analysis (Table 6), thus demonstrating the potential usefulness of the series for drug-like compound growth.

#### 3.5.5. Medicinal Chemistry

Pan-assay interference compounds or promiscuous compounds (PAINS) are substances with substructures that display a false reaction, irrespective of the protein receptor, with biologically potent output [46]. Table 6 shows that all of the compounds return no PAINS alert. For all the candidates in the library, the synthetic accessibility (SA) scores were found to be less than 5 except for the 6 compounds that had scores more than 5, as shown in Table 6. The score of SA is normalized between 1 (easy synthesis) and 10 (very difficult synthesis). For most candidates in the library, the SA scores were found to be less than 5 and thus have strong synthesis feasibility.

The lead-like rule-based approach lets the medicinal chemist define the required molecule to start lead optimization. Interestingly, Table 6 shows that 10 compounds of 26 have one violation of lead-likeness, and these molecules are therefore considered appropriate for initiating lead optimization. Furthermore, as is evident from the radar depictions, 8 compounds with two violations are also found to meet the requirement for oral bioavailability.

#### 3.5.6. Bioavailability Radar

**Figure 2:**
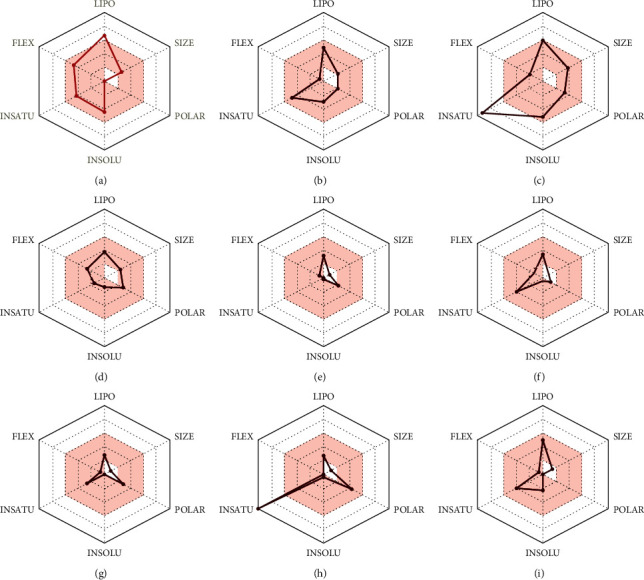
Structures of bioavailability radar (a): 1,6,10-dodecatriene, 7,11-dimethyl-3-methylene, (b) 2-phenylthiolane propanamide, (c) phthalonitrile, (d) cyclohexane propanoic acid, (e) cyclopropane tetradecanoic acid, (f) N-methylallylamine, (g) propanamide, (h) 1H-tetrazol-5-amine, and (i) cyclohexene, 3-ethenyl.

The drug-likeness of a molecule can be quickly determined using the bioavailability radar. The pink-colored region is the required physiochemical space for oral bioavailability, and the molecule's radar plot must fall entirely into the field to be considered drug-like [47]. The SwissADME prediction output revealed that 9 compounds had the optimal range of all six properties, which indicates that they have competent chemotherapeutic potential (Figure 2).

#### 3.5.7. Molecular Docking

The molecular docking technique is used to calculate ligand-binding affinities and energies, which is essential in the structure-based drug design process. 3D protonation, energy minimization, and prediction of the active site for ligands were used to prepare the protein for molecular docking, with the parameters left at their defaults. Then, using GOLD version 5.3.0 software, ligands were docked with the target protein (5 kzw). In our study, 9 secondary metabolites from *T. divaricata* (Jasmine) flower were docked with *α*-glucosidase protein (5 kzw), which is the important protein for the key regulatory enzymes that are important in the diabetes management. To prevent sugar digestion and postprandial hyperglycemia, glucosidase, a digestive enzyme involved in carbohydrate digestion, must be inhibited.  Table 7 contains a list of the compounds, which are downloaded from PubChem (https://pubchem.ncbi.nlm.nih.gov).

Following the selection of these nine compounds based on SwissADME findings, ChemDraw Ultra 12.0 and Chem3D Pro were used in GOLD docking for energy minimization of ligands. The coordinate crystal structure of the 5kzw protein was obtained from the Protein Data Bank and loaded into GOLD suite version 5.3.0 with a resolution of 0. GOLD 5.3.0 version was used to screen various docked complexes based on docking fitness and GOLD ratings. The GOLD program identified the most effective compound for interacting with the receptor of 2.70. The binding compatibility, i.e., docking score and fitness, was used to test the results. The best drug was selected with the highest binding affinity with the receptor molecule. As the ligand molecule, the number of hydrogen bonds formed and the bond distance between the active site and inhibited atomic coordinates were used to decide the final docked conformation for various chemicals. The GOLD docking scores for the phytochemicals are given in Table 8.

For each ligand, each docking routine returned the top ten rated docked poses. Compounds having maximum ligand-receptor binding energy and interactions with the receptor (<6 Å bond lengths) were predicted to be most effective. Cyclopropane tetradecanoic acid, 2-octyl-, methyl ester, 4-(4-methyl-2-biphenylyloxy) phthalonitrile showed the best interaction with *α*-glucosidase (PDB ID = 5kzw), and the protein having Gold fitness (39.28, 37.21) and GOLD docking score is -7.51and -6.53 including forming a hydrogen bond with LEU A: 777, ARG A: 779, ASN A: 780, THR A: 782, VAL A: 784, LEU A: 777, ARG A: 779, VAL A: 784, LEU A: 815, SER A: 848, TRP A: 849, CYS A: 850, and LEU A: 846, respectively. These molecules showed exceptionally good interaction with *α*-glucosidase protein. 1,6,10-dodecatriene, 7,11-dimethyl-3-methylene, 2-phenylthiolane, and cyclohexane propanoic acid, 3-oxo-, methyl ester showed moderate binding affinity (32.69, 29.14, and 28.29, respectively), and GOLD docking score is -4.03, 0.205, and 0.93, respectively, having the interaction of hydrogen bond with LEU A: 777, ARG A: 779, VAL A: 784, LEU A: 777, ARG A: 779, VAL A: 784, LEU A: 846, ARG A: 779, and VAL A: 784. Compounds (N-methylallylamine, propanamide, and cyclohexene, 3-ethenyl) had the least interaction in the range of 20.00, 19.16, and 23.83 with docking scores of -0.16, -0.59, and 0.14.1H-Tetrazol-5-amine compound showed least interactions with the range of fitness of 17.21 and docking score of 0.00.

**Figure 3:**
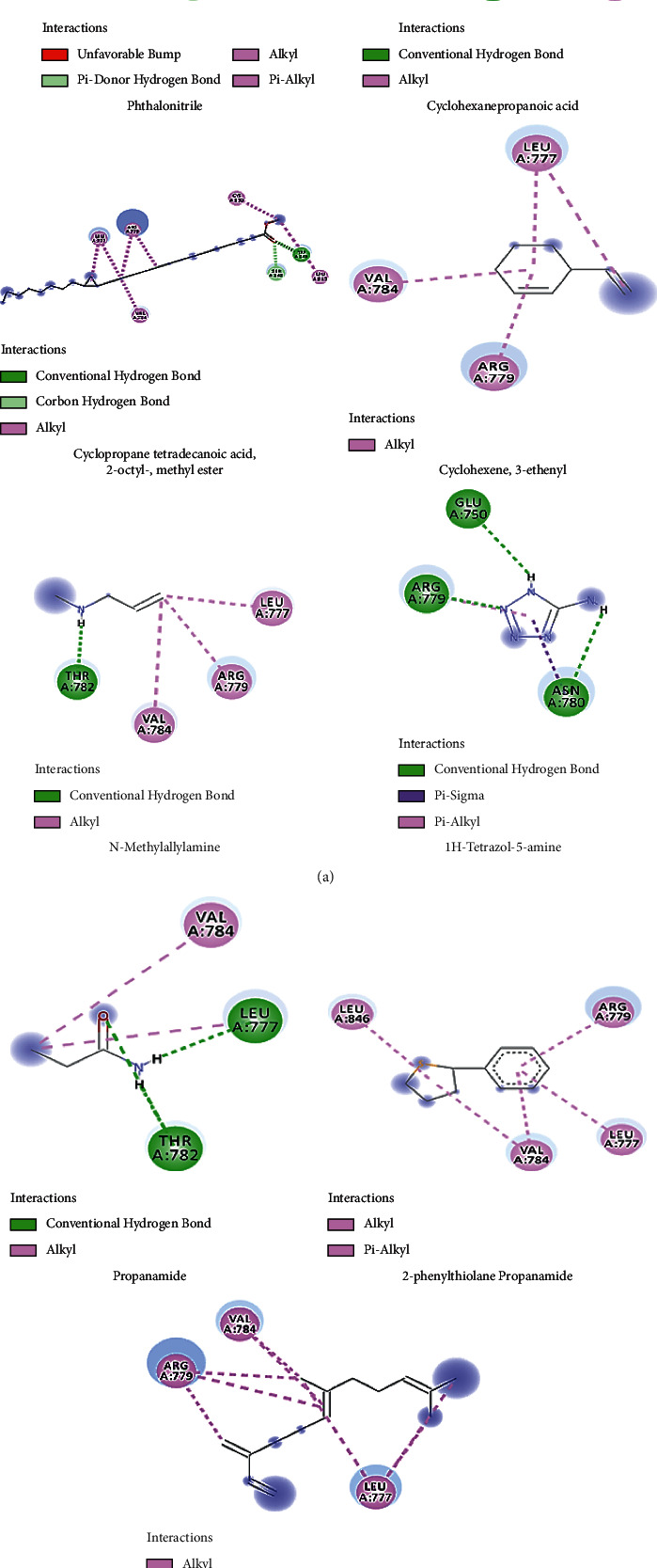
*In silico* molecular docking results of interactions between 5kzw proteins with screened compounds as ligand.

2D view of protein-ligand interactions from the best poses produced by Discovery Studio showed that all molecules exhibited the same binding mode, as shown in Figure 3. ARG A: 779, LEU A: 777, and VAL A: 784 are three important interactions between these atoms and the residues.

## 4. Discussion

Ethnobotanicals are widely used for the treatment of diabetic and oxidative stress-related conditions [48]; however, it still requires rigorous scientific validation. The amount of postprandial blood glucose is the key factor that must be controlled in T2D management [49]. The drug intervention often has inexorable consequences, mostly hypoglycemia, gastrointestinal damage, and weight gain [50]. Several studies have verified the antihyperglycemic properties of plant extracts and herbal formulations that could be used as antidiabetic tonics. Herbal medications are often thought to have fewer adverse side effects than prescription drugs [51].

The correlation was found between hypoglycemic events of medicinal plants to the presence of phenol and flavonoids [52]. According to previous studies, flavonoids have an antidiabetic property and are a source of glucose uptake in tissue with relevance to oxidative stress during diabetic conditions [53]. Antidiabetic activity is due to the co-adjuvant effect of bioactive compounds present in *T. divaricata*.

Plants have a phenolic compound that shows antioxidant activity to prevent tissue damage. Rauter et al. [53] reported phenolic content as antioxidants in selected Nigerian medicinal plants, such as *A. platyneuron* and *B. nitida*, in which the value of phenolic content ranged from 82.33 ± 0.30 to 11.67 ± 0.09 mg GAE/g, while Agbo et al. [54] reported that the phenolic content ranged from 97.77 ± 0.77 mg GAE/g. In our results, *T. divaricate* showed maximum phenolic content of 62.32 ± 4.02. Flavonoid is another parameter that is used for the determination of antioxidant activity in medicinal plants. In this study, the average range of flavonoid ranged from 13.95 ± 1.33 to 24.53 ± 0.61. Agbo et al. [54] reported flavonoid values in *M. afzelii* extract, i.e., 3.67 ± 0.00 mg QE/g, while Saeed et al. [55] reported the value of 59.6 ± 1.5 for methanolic plant extract of *T. leptophylla*.

Previous research has shown that diabetic beta cells produce reactive oxygen species (ROS), which are counteracted by the overexpression of antioxidant enzymes such as superoxide dismutase (SOD), catalase (CAT), and peroxidase (POX). Antioxidants protect beta cells from oxidative damage by scavenging the free radicals produced, thus preventing diabetes from developing [27–29]. In transgenic mice, overexpression of SOD has been shown to prevent the development of diabetic complications. Since oxidative stress is linked to the development of diabetic complications and inflammation, this study aimed to assess *Tabernaemontana divaricata's* antioxidant activity.

Biological properties, such as the antioxidant property, are considered as an estimation of the nutritional and medicinal value of plants [56]. The antioxidant activity of methanolic extract of *T. divaricata* flowers was estimated by DPPH assay. DPPH is a neutral, free radical that helps a stable molecule to tolerate an electron or hydrogen radical. The ability of natural antioxidants to reduce the DPPH free radical is assessed by a decrease in absorbance at 520 nm. In comparison with regular ascorbic acid, the extract showed strong scavenging activity. The lower the absorbance, the more the scavenging operation there was. As a result, the bioactive compounds in this plant can serve as antioxidants and aid in the treatment of a variety of diseases, including diabetes. Antioxidant properties of natural compounds are said to correlate with antidiabetic properties or vice versa. In this study, the given antioxidant activity of the *T. divaricata* flower extract could be connected to their good amount of TFC and TPC, which function as metal chelators, reduction agents, hydrogen donors, and singlet oxygen quenchers and free radical scavenger [57].

Toxicity is an important factor in the design of pharmaceutical drugs and an important starting point for hemolytic actions, providing principal knowledge on the interaction at the cellular level between biological entities and molecules. Hemolytic activity of any compound is a sign of common cytotoxicity towards normal healthy cells. In this research, MEAE exhibited 10% cytotoxicity toward human erythrocytes, as shown in Table 3. Such findings demonstrate that if medicinal formulations from this plant are used at low concentrations, parameters of the RBC membrane will not change. Reports on the toxic effects of *A. esculentus* are insufficient. A previous study conducted with aqueous and methanolic extracts of the *A. esculentus* fruit confirmed that there were no deaths up to a dose of 2000 mg/kg (p.o.) in Swiss mice (*n* = 6) for 7 days and no signs of toxicity [58], which may be one of the significant issues for considering it as an important vegetable for human use. Further research is essential on this medicinal edible plant in the context of drug development and discovery.

Dietary phenols and flavonoids, in accumulation to theirantioxidant effects, have been noticed to exert antihyperglycemic effects by binding to the transporters of glucose [49] and spirited digestive enzyme inhibition [59]. The carbohydrate-digesting enzymes, *α*-glucosidase and *α*-amylase, digest dietary starch and produce glucose, resulting in surge of postprandial glucose. So, the inhibition of *α*-glucosidase and *α*-amylase activities is one of the primary approaches to managing hyperglycemic T2D patients.

*T. divaricata* has antidiabetic activity, according to alpha-amylase inhibitory tests. The percentage inhibitory activity of plant methanolic extract against alpha-amylase enzyme increased in a dose-dependent manner. The plant extract concentration of 50 g/ml showed a percentage inhibition of 28%, while 200 g/ml showed a percentage inhibition of 61%. At a high concentration of 200 g/ml, the extract inhibited the alpha-amylase enzyme by 50%. As a result of the current inhibitory studies, it has been discovered that the *T. divaricata* plant extract is successful in inhibiting the alpha-amylase enzyme, which helps to postpone the breakdown of starch into glucose and thus maintain glucose levels in diabetic patients. Dastjerdi et al. [48] observed *α*-amylase inhibition activity of some plant extracts of *T. eucrium species* values ranged from 41.59 ± 0.64 to 77.07 ± 0.49 at 1.56 mg/mL to 25 mg/mL. The *α*-amylase inhibitory activity of methanol plant extract is most likely by polar compounds that should be investigated further by isolating pure active compounds.

At 50–200 g/ml concentrations of *T. divaricata* extract, the percentage inhibition increased in a dose-dependent manner. For the highest and lowest concentrations, respectively, the percentage inhibition ranged from 60% to 31% (Table 4). Thus, by inhibiting the alpha-glucosidase enzyme, the extract of *T. divaricata,* which contains various natural bioactive compounds, helps to reduce the rate of carbohydrate digestion, lowering blood glucose levels and maintaining diabetic conditions.

Glucose uptake is one of the in vitro methods used to explore the antihyperglycemic effect of different compounds [60]. The average values of glucose uptake by yeast cells in this study were 13.05 ± 1.3% to 69.5 ± 4.78%. Shehzadi et al. [60] reported the evaluation of antihyperglycemic activity through in vitro assays (0.2 to 26.3%) at 2 mg/mL, which was below the current research values. The results indicated all used concentrations under examination are capable of increasing the utilization and uptake of glucose in yeast cells.

The *in silico* prediction of molecular physicochemical parameters, bioavailability, and the pharmacokinetics have become more relevant for the investigation of productive potential drug molecules from a drug discovery standpoint [47]. Theoretical experiments play a critical role in presenting accurate data in a timely and comfortable manner. Many free online platforms have recently been established for faster screening, reducing the time and expense of drug testing (no animal testing) [46]. SwissADME is a modern comprehensive method developed by the Swiss Institute of Bioinformatics (SIB) that allows drug candidates to have their absorption, delivery, metabolism, and excretion (ADME) parameters estimated [45]. At the outset of the drug development process, ADME properties, which determine either the potential drug's access to the target or its removal by the organism, are the required properties that must be evaluated for the drug to be selected. In silico experiments based on measured physicochemical requirements can be used to check these parameters. SwissADME also provides information on gastrointestinal absorption (GIA) and blood-brain barrier (BBB) permeability in humans. From twenty-six selected compounds, only eight compounds fulfilled the criteria of drug-likeness parameters. So, all of these nine compounds were further processed for molecular docking by GOLD.

To predict the affinity and behavior of small molecules and drug candidates, computational molecular docking is commonly used to predict the binding orientation to their protein targets [61]. We used molecular docking to model various bioactive compounds isolated from the Jasmine plant that is known to inhibit glucosidase. Because of their low price and comparatively greater protection, with a low frequency of severe gastrointestinal side effects, plants or plant-based substances may be a suitable source of glucosidase inhibitors [62]. Hydrogen bonds play a crucial role in the structure and interaction of protein-protein or ligand-receptor complexes. Hydrogen bonds are important in drug design to ensure that the drug is unique to the protein target. To back up the findings of this research, a molecular docking study was performed (cyclopropane tetradecanoic acid, 2-octyl-, methyl ester, 4-(4-methyl-2-biphenylyloxy) phthalonitrile), which showed excellent interaction with 5kzw protein having Gold fitness (39.28, 37.21) and GOLD docking score of -7.51 and -6.53 including the formation of hydrogen bond with amino acids residues (1,6,10-dodecatriene, 7,11-dimethyl-3-methylene, 2-phenylthiolane, and cyclohexane propanoic acid, 3-oxo-, methyl ester). These molecules showed exceptionally good interaction with *α*-glucosidase protein and can be considered as potential molecules that may prove to be beneficial in antiviral activity through their direct action on new castle disease [63]. These compounds showed moderate binding affinity of 32.69, 29.14, and 28.29 and GOLD docking scores of -4.03, 0.205, and 0.93, respectively. N-methylallylamine, propanamide, cyclohexene, and 3-ethenyl have the least interaction in the range of 20.00, 19.16, and 23.83 and docking scores of -0.16, -0.59, and 0.14, while 1H-tetrazol-5-amine compound showed the least affinity towards 5kzw binding site.

## 5. Conclusion

Since diabetes mellitus is a global epidemic, effective drugs with less or no toxicity must be developed and one such way is the use of herbal medicines having no side effects. This study investigated the antidiabetic and antioxidant properties of *T. divaricata* extract and found promising inhibitors of two carbohydrate-related enzymes. *T. divaricata* has rich phenolic and flavonoid contents. As a flavonoid, it has great antidiabetic potential and also causes active uptake of glucose. The methanolic extract of *T. divaricate* has great potential for stimulation of *α*-amylase and *α*-glucosidase, so that it can be used to treat diabetes. *In silico* docking studies of phytoactive compounds from *T. divaricata* have been proven to have potential drug capabilities in terms of their pharmacokinetic and drug-likeness. The molecular docking analysis described some compounds that inhibited the targets related to diabetes mellitus and showed encouraging results with prominent inhibitory activity. This study shows that *T. divaricata* extract, which is rich in important bioactive compounds, can be used for diabetes. It is suggested to carry out long-term research work to recognize and isolate the active moieties responsible for antidiabetic property and to understand the mechanisms involved in glucose-lowering properties of the plant.

## Figures and Tables

**Table 1:** Physicochemical properties of flower extract of *T. divaricate.*

Physicochemical properties											
Compound	Formula	MW	Heavy atom	Arom HA	Csp3	Rotatable bond	H+ bond acceptor	H+ bond donor	Molar refractivity	TPSA	Lipophilicity Conseco log Po/w
Dodecatriene	C1_5_H_24_	204.35 g\mol	15	0	0.47	7	0	0	72.32	0.00	3.86
Cyclohexane propanoic acid	C_10_H_16_O_3_	184.23 g\mol	13	0	0.80	4	3	0	49.55	43.37	2.13
D-Glucopyranoside	C_18_H_32_O_16_	504.44 g\mol	34	0	1.00	8	16	11	100.54	268.68	−0.64
Vitamin D3	C_27_H_44_O	384.64 g\mol	28	0	0.78	6	1	1	125.04	20.23	5.08
Desulphosinigrin	C_10_H_17_NO_65_	279.31 g\mol	18	0	0.70	5	7	5	65.33	148.04	0.85
Lactose	C_12_H_44_O_11_	342.30 g\mol	23	0	1.00	4	11	8	68.12	189.53	1.35
n-Hexadecanoic acid	C_16_H_32_O_2_	256.42 g\mol	18	0	0.94	14	2	1	80.80	37.30	3.85
Cyclopropane tetradecanoic acid	C_26_H_50_O_2_	394.67 g\mol	28	0	0.96	22	0	2	126.27	26.30	6.48
Octadecadienoic acid	C_18_H_32_O_2_	280.45 g\mol	20	0	0.72	14	2	1	89.46	37.30	4.14
Phthalonitrile	C_21_H_14_N_2O_	310.35 g\mol	24	18	0.05	3	3	0	92.79	56.81	3.03
Octadecatrienoic acid	C_25_H_40_O_6_	436.58 g\mol	31	0	0.64	21	6	0	124.72	78.90	4.97
Squalene	C_30_H_50_	410.72 g\mol	30	0	0.60	15	0	0	143.48	0.00	6.37
Cholestan	C_28_H_48_O	400.58 g\mol	29	0	0.93	5	1	1	128.42	20.43	5.02
Vitamin A aldehyde	C_20_H_28_O	284.44 g\mol	21	0	0.45	5	1	0	93.71	17.07	3.96

**Table 2:** Phytochemical constituents and antioxidant activity of methanolic extract of *T. divaricate.*

Concentration of extract	Total phenol (mg/g)	Total flavonoid (mg/g)	%Inhibition by DPPH	%Inhibition by ascorbic acid
1 mg	62.32 ± 4.02	24.53 ± 0.61	15.9 ± 2.33	65.57 ± 13.4
500 *μ*g	55.11 ± 6.74	22.25 ± 2.00	6.74 ± 1.45	43.34 ± 31.4
250 *μ*g	44.37 ± 8.48	17.82 ± 1.42	4.23 ± 0.33	7.51 ± 2.08
125 *μ*g	41.36 ± 7.46	15.03 ± 1.06	2.50 ± 0.33	2.50 ± 0.33
62 *μ*g	39.15 ± 5.94	14.72 ± 1.55	2.88 ± 0.57	1.73 ± 0.58
31 *μ*g	35.62 ± 5.31	13.95 ± 1.33	1.49 ± 0.33	1.15 ± 0.58

**Table 3:** Different enzymatic activities and cytotoxicity of methanolic extract of *T. divaricata* flower.

	Enzymatic assays (U/mL protein)			Cytotoxicity %
	CAT	POD	SOD	
*T. divaricata*	3.02 ± 3.4	0.74 ± 0.2	15.87 ± 0.5	10
-Ve control	2.89 ± 0.11	0.51 ± 0.1	2.75 ± 0.35	0.63
Triton X	—	—	—	96

**Table 4:** Percentage inhibition of *α*-amylase and *α*-glucosidase of *T. divaricate.*

Extract concentration	%Inhibition of *α*-amylase	% Inhibition of *α*-glucosidase
200 *μ*g/ml	41.81 ± 3.75	35.9 ± 1.24
150 *μ*g/ml	35.78 ± 4.12	31.78 ± 1.72
100 *μ*g/ml	26.1 ± 4.01	22.71 ± 3.11
50 *μ*g/ml	16.76 ± 3.97	17.88 ± 1.40

**Table 5:** Pharmacokinetic properties of flower extract of *T. divaricate.*

Pharmacokinetics									
Compound	Gi absorption	BBB permeant	P-gp substrate	CYP1A2 inhibitor	CYP2C19 inhibitor	CYP2C9 inhibitor	CYP2D6 inhibitor	CYP3A4 inhibitor	Log Kp (skin permeation)
Dodecatriene	low	No	No	Yes	No	Yes	No	No	−3.27
Cyclohexane propanoic acid	High	Yes	No	No	No	No	No	No	−6.70
D-Glucopyranoside	Low	No	Yes	No	No	No	No	No	−13.53
Vitamin D3	Low	No	No	No	No	Yes	No	No	−3.00
Desulphosinigrin	Low	No	Yes	No	No	No	No	No	−8.91
Lactose	Low	No	Yes	No	No	No	No	No	−10.92
n-Hexadecanoic acid	High	Yes	No	Yes	No	Yes	No	No	−2.77
Cyclopropane tetradecanoic acid	Low	No	Yes	Yes	No	No	No	No	−0.75
Octadecadienoic acid	High	Yes	No	Yes	No	Yes	No	No	−3.05
Phthalonitrile	High	Yes	No	Yes	Yes	Yes	No	No	−4.73
Octadecatrienoic acid	High	No	Yes	No	Yes	Yes	No	Yes	−4.65
Squalene	Low	No	No	No	No	No	No	No	−0.58
Cholestan	Low	No	No	No	No	No	No	No	−2.25
Vitamin A aldehyde	High	No	No	Yes	No	Yes	No	No	−3.60
1-Heptatriacotanol	Low	No	Yes	No	No	No	No	No	3.55
Methyl ester	Low	No	Yes	Yes	No	No	No	No	−3.31
Acetaldehyde	Low	No	Yes	Yes	No	No	No	No	−6.76
2-Phenylthiolane	High	Yes	No	Yes	No	No	No	No	−5.25
Propanamide	High	No	No	No	No	No	No	No	−7.21
Cyclopropane	High	No	No	No	No	No	No	No	−6.71
Cyclohexene, 3-ethenyl	Low	Yes	No	No	No	No	No	No	−4.78
N-Methylallylamine	Low	No	No	No	No	No	No	No	−6.42
Tetrazole	High	No	No	No	No	No	No	No	−7.44
Benzene dicarboxylic acid	High	No	No	No	No	No	No	No	−2.71
Methylnonadecane	Low	No	No	Yes	No	No	No	No	−0.38
Phthalic acid	Low	No	No	No	No	No	No	Yes	−3.16

**Table 6:** Drug-likeness and medicinal properties of flower extract of *T. divaricate.*

Drug-likeness							Medicinal chemistry		
Compound	Lipinski	Ghose	Veber	Egen	Muegge	Bioavailability score	PAINS	Leadlikeness	Synthetic accessibility
Dodecatriene	Yes; 1 violation	Yes	Yes	Yes	No; 2 violation	0.55	0 alert	No; 2 violation	3.42

Cyclohexane propanoic acid	Yes; 0 violation	Yes	Yes	Yes	No; 1 violation	0.55	0 alert	No; 1 violation	2.20

D-Glucopyranoside	No; 3 violation	No; 2 violation	No; 1 violation	No; 1 violation	No; 4 violation	0.17	0 alert	No; 2 violation	6.23

Vitamin D3	No; 1 violation	No; 2 violation	Yes	No; 1 violation	No; 2 violation	0.55	0 alert	No; 2 violation	6.02

Desulphosinigrin	Yes; 0 violation	No; 1 violation	No; 1 violation	No; 1 violation	Yes	0.55	0 alert	Yes	5.14

Lactose	No; 2 violation	No; 1 violation	No; 1 violation	No; 1 violation	No; 4 violation	0.17	0 alert	Yes	5.41

n-Hexadecanoic acid	Yes; 1 violation	Yes	No; 1 violation	Yes	No; 1 violation	0.85	0 alert	No; 2 violation	2.31

Cyclopropane tetradecanoic acid	Yes; 1 violation	No; 2 violation	No; 1 violation	No; 1 violation	No; 2 violation	0.55	0 alert	No; 3 violation	4.30

Octadecadienoic acid	Yes; 1 violation	No; 1 violation	No; 1 violation	No; 1 violation	No; 1 violation	0.85	0 alert	No; 2 violation	3.10

Phthalonitrile	Yes; 0 violation	Yes	Yes	Yes	Yes	0.55	0 alert	No; 1 violation	2.73

Octadecatrienoic acid	Yes; 0 violation	No; 2 violation	No; 1 violation	Yes	No; 2 violation	0.55	0 alert	No; 3 violation	4.54

Squalene	Yes; 1 violation	No; 3 violation	No; 1 violation	No; 1 violation	No; 2 violation	0.55	0 alert	No; 3 violation	4.73

Cholestan	Yes; 1 violation	No; 2 violation	Yes	No; 1 violation	No; 2 violation	0.55	0 alert	No; 2violation	5.30

Vitamin A aldehyde	Yes; 1 violation	No; 1 violation	Yes	Yes	No; 2 violation	0.55	0 alert	No; 1 violation	4.16

1-Heptatriacotanol	No; 2 violation	No; 4 violation	No; 1 violation	No; 1 violation	No; 3 violation	0.17	0 alert	No; 3 violation	4.87

Methyl ester	Yes; 1 violation	No; 3 violation	Yes	No; 1 violation	No; 1 violation	0.55	0 alert	No; 2 violation	6.04

Acetaldehyde	Yes; 0 violation	No; 3 violation	Yes	Yes	No; 3 violation	0.55	0 alert	No; 1 violation	1.00

2-Phenylthiolane	Yes; 0 violation	Yes	Yes	Yes	No; 2 violation	0.55	0 alert	No; 1 violation	2.41

Propanamide	Yes; 0 violation	No; 3 violation	Yes	Yes	No; 2 violation	0.55	0 alert	No; 1 violation	1.00

Cyclopropane	Yes; 0 violation	No; 3 violation	Yes	Yes	No; 3 violation	0.55	0 alert	No; 1 violation	1.00

Cyclohexene, 3-ethenyl	Yes; 0 violation	No; 2 violation	Yes	Yes	No; 2 violation	0.55	0 alert	No; 1 violation	3.20

N-Methylallylamine	Yes;0 violation	No; 3 violation	Yes	Yes	No; 3 violation	0.55	0 alert	No; 1 violation	1.02

Tetrazole	Yes; 0 violation	No; 4 violation	Yes	Yes	No; 2 violation	0.56	0 alert	No; 1 violation	2.12

Benzene dicarboxylic acid	Yes; 1 violation	No; 1 violation	No; 1 violation	No; 1 violation	No; 2 violation	0.55	0 alert	No; 3 violation	3.41

Methylnonadecane	Yes; 1 violation	No; 1 violation	No; 1 violation	No; 1 violation	No; 3 violation	0.55	0 alert	No; 2 violation	2.72

Phthalic acid	Yes; 1 violation	No; 2 violation	No; 1 violation	No; 1 violation	No; 2 violation	0.55	0 alert	No; 3 violation	3.65

**Table 7:** Nine selective compound properties of molecular formula/weight for docking.

Name of the compound	Molecular formula	MW	PubChem ID	Structure
1,6,10-Dodecatriene, 7,11-dimethyl-3-methylene-, (Z)-	C15H24	204	5317319	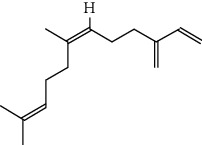
Cyclohexane propanoic acid, 3-oxo-, methyl ester	C10H16O3	184	538977	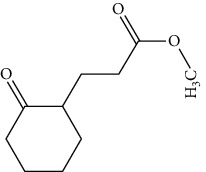
Cyclopropane tetradecanoic acid, 2-octyl-, methyl ester	C26H50O2	394	552099	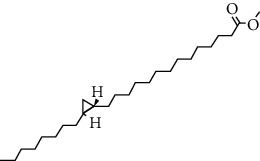
4-(4-Methyl-2-biphenylyloxy) phthalonitrile	C21H14N2O	310	628209	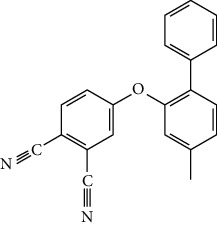
2-Phenylthiolane	C10H12S	164.27	596288	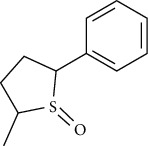
Propanamide	C3H7NO	73.09	6578	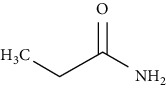
Cyclohexene, 3-ethenyl	C8H12	108.18	522623	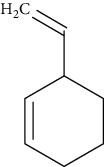
N-Methylallylamine	C4H10ClN	107.58	18326499	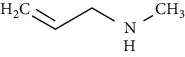
1H-Tetrazol-5-amine	<CH5N5O	103.08	12211273	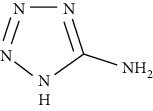

**Table 8:** Docking interactions of different active compounds of *T. divaricate.*

Compound	Gold score	Gold binding fitness	RMSD	Residue	Hydrophobicity	Pka	Distance
2-phenylthiolane	0.205	29.14	78.61	LEU777	3.8		4.76
				ARG779	−4.5	12	3.84
				VAL784	4.2		4.92
				LEU846	3.8		5.09
4-(4-Methyl-2-biphenylyloxy) phthalonitrile	−6.53	37.21	69.18	LEU777	3.8		4.57
				ARG779	−4.5	12	4.7
				ASN780	−3.5		3.19
				THR782	−0.7		2.34
				VAL784	4.2		5.08
				LEU846	3.8		2.20
Cyclohexanepropanoic acid, 3-oxo-, methyl ester	−0.93	28.29	65.43	ARG779	−4.5	12	2.26
				VAL784	4.2		4.42
Cyclopropane tetradecanoic acid, 2-octyl-, methyl ester	−7.51	39.28	69.09	LEU777	3.8		5.32
				ARG779	−4.5	12	4.61
				VAL784	4.2		5.02
				LEU815	3.8		5.08
				SER848	−0.8		2.47
				TRP849	−0.9		2.64
				CYS850	2.5	9	3.74
1,6,10-Dodecatriene, 7,11-dimethyl-3-methylene-	−4.03	32.69	67.03	LEU777	3.8		5.13
				ARG779	−4.5	12	4.99
				VAL784	4.2		3.88
N-Methylallylamine	−0.16	20.00	65.88	LEU777	3.8		4.08
				ARG779	−4.5	12	3.70
				THR782	−0.7		1.52
				VAL784	4.2		4.84
Propanamide	−0.59	19.16	65.42	LEU777	3.8		2.20
				THR782	−0.7		2.86
				VAL784	4.2		5.28
1H-Tetrazol-5-amine	0.00	17.21	66.78	GLU750	−3.5	4.3	2.59
				ARG779	−4.5	12	1.85
				ASN780	−3.5		2.74
Cyclohexene, 3-ethenyl	0.14	23.83	64.50	LEU777	3.8		4.97
				ARG779	−4.5	12	3.89
				VAL784	4.2		4.35

## Data Availability

Data are available from authors on reasonable demand.
